# Epidemiology of Patent Foramen Ovale in General Population and in Stroke Patients: A Narrative Review

**DOI:** 10.3389/fneur.2020.00281

**Published:** 2020-04-28

**Authors:** Ioanna Koutroulou, Georgios Tsivgoulis, Dimitrios Tsalikakis, Dimitris Karacostas, Nikolaos Grigoriadis, Theodoros Karapanayiotides

**Affiliations:** ^1^Second Department of Neurology, AHEPA University Hospital, Aristotle University of Thessaloniki, Thessaloniki, Greece; ^2^Second Department of Neurology, Attikon Hospital, National and Kapodistrian University of Athens, Athens, Greece; ^3^Polytechnic School, University of Western Macedonia, Kozani, Greece

**Keywords:** PFO, epidemiology, stroke, TCD, review

## Abstract

**Introduction:** Percutaneous closure of patent foramen ovale (PFO) in selected patients with cryptogenic cerebrovascular ischemic events (CEs) decreases the risk of recurrent stroke; however, optimal patient selection criteria are still under investigation. Candidates for PFO closure are usually selected from the pool of CE patients with a high risk of Paradoxical Embolism (RoPE) score. The RoPE score calculates the probability that PFO is causally related to stroke, based on PFO prevalence in patients with CE compared with that in healthy subjects. The latter has been set at 25% based on the average of autopsy and transesophageal echocardiography (TEE) studies.

**Methods:** We conducted a comprehensive review of studies investigating PFO prevalence in general population and in patients with CE and non-CE using autopsy, TEE, transcranial Doppler (TCD) or transthoracic echocardiography (TTE). Studies were excluded if they (1) reported data from referred subjects with underlying cerebrovascular disease or (2) did not specify etiologically the events.

**Results:** In healthy/control subjects, PFO prevalence was 24.2% (1,872/7,747) in autopsy studies, 23.7% (325/1,369) in TEE, 31.3% (111/355) in TCD, and 14.7% (186/1,267) in TTE studies. All diagnostic modalities included PFO prevalence was higher in CE compared with healthy/control population [odds ratio (OR) = 3.1, 95% confidence interval (CI) = 2.5–3.8] and compared with non-CE (OR = 2.3, 95% CI = 2.0–2.6). In patients with CE, PFO prevalence in the young compared to the old was higher when the diagnostic modality was TEE (48.9 vs. 27.3%, *p* < 0.0001, OR = 2.6 with 95% CI = 2.0–3.3) or TCD (58.1 vs. 41%, OR = 1.9, 95% CI = 1.6–2.5), but not TTE (53.3 vs. 37.5%, *p* = 0.16). Regarding non-CE, PFO prevalence in the young compared to the old was higher when the diagnostic modality was TEE (20 vs. 12.9%, OR = 1.7, 95% CI = 1.0–2.8) but not TTE (10.4 vs. 7.8%, *p* = 0.75) or TCD (22.8 vs. 20.1%, *p* = 0.56).

**Conclusions:** Given the limitations of autopsy and TEE studies, there is good reason not to take a fixed 25% PFO prevalence for granted. The estimation of degree of causality may be underestimated or overestimated in populations with PFO prevalence significantly lower or higher than the established. Given the high sensitivity, non-invasive nature, low cost, and repeatability of TCD, future large-scale TCD-based studies should investigate potential heterogeneity in PFO prevalence in different healthy racial/ethnic populations.

## Introduction

In 1564, the Italian anatomist and surgeon Leonardo Botallo claimed in his publication “De catarrho commentarius” that he had discovered a “duct,” which connected the right with the left atrium. He called it the “vena arteriarum nutria,” which is nowadays known as foramen ovale or foramen Botalli (1). Three centuries later, Julius Cohnheim, a German professor of pathology, was the first to describe a case of fatal paradoxical embolism through a patent foramen ovale (PFO) to the middle cerebral artery (2). In 1880, Moritz Litten documented a second case of paradoxical embolism to the lower extremity (2). Patency of the foramen ovale is normal during fetal life allowing blood from the inferior vena cava to pass from the right to the left atrium, bypassing the lungs. At birth, pulmonary blood flow increases greatly because right heart pressure and pulmonary vascular resistance drop as pulmonary arterioles open in reaction to oxygen filling the alveoli. Left atrial pressure is increased resulting in functional closure of the foramen ovale. Anatomic closure occurs later in infancy in the majority of population, but sometimes the closure is incomplete and remains as PFO (3, 4).

Despite a thorough investigation, the etiology of cerebrovascular ischemic events remains undetermined in almost 10–40% of cases (5). Numerous case-control studies showed that PFO prevalence is remarkably high in patients with cryptogenic strokes (CSs) compared to the healthy population. It is considered that a part of these strokes may be attributed to paradoxical embolism or *in situ* thrombus formation in a PFO niche; therefore, PFO closure may be effective in secondary stroke prevention. The first three randomized controlled trials (RCTs) that addressed this issue (CLOSURE I, RESPECT, PC Trial) (6–8) failed to show superiority of PFO closure vs. best medical treatment (9). Despite the negative results, the suspicion that PFO was etiologically related with CS was strong. Four years later, three new RCTs (CLOSE, Gore REDUCE, DEFENSE-PFO) (10–12) and the extended follow-up results of the RESPECT trial (13) showed superiority of PFO closure compared to antiplatelet agents in appropriately selected patients using specific devices (14). Nevertheless, the optimal candidates for PFO closure are still not precisely known. The Risk of Paradoxical Embolism (RoPE) score (15) has been developed to facilitate the selection of CS patients who might benefit from PFO closure. The RoPE score applies Bayes' theorem and calculates the probability that PFO is causally related to stroke [PFO attributable fraction (PFOAF)], with higher scores implying greater possibility that a PFO is etiologically associated with a CS. Calculations are based on PFO prevalence in patients with CS compared with that in healthy subjects. The latter is considered to be 25% and the former is estimated at 40%, based on the RoPE database of 3,674 patients with CS (15). However, PFO prevalence in non-selected populations varies widely, and PFOAF may be “inflated” or “deflated,” depending on numbers.

Therefore, we conducted a comprehensive critical review of the available epidemiological data on PFO prevalence in the general population and in stroke (cryptogenic and non-cryptogenic) stratified by diagnostic modality [autopsy, transthoracic (TTE) and transesophageal echocardiography (TEE), transcranial Doppler (TCD)] and by age (young vs. old). We provide a critical appraisal of each PFO screening modality, and we underscore methodological downsides of individual epidemiological studies that have impacted on the estimation of PFO prevalence in the general population and in distinct stroke patient subgroups and hitherto have been uncommented on.

## Methods

We performed a detailed search in MEDLINE, SCOPUS, Cochrane Library, and Google scholar up to November 1, 2019, using the following terms in combination: “patent foramen ovale,” “PFO,” “right-to-left-shunt,” “prevalence of patent foramen ovale,” “prevalence of PFO,” “frequency of PFO,” “cryptogenic stroke,” “cryptogenic stroke and patent foramen ovale,” “autopsy studies and patent foramen ovale,” “transthoracic echocardiography and patent foramen ovale,” “transesophageal echocardiography and patent foramen ovale,” “transcranial Doppler and patent foramen ovale,” “PFO and cerebrovascular ischemic events,” “PFO and migraine.” We also searched the reference lists of all relevant articles. Both English and foreign language articles were reviewed. We included case-control, population-based, and cohort studies that examined PFO prevalence in patients with cerebrovascular ischemic events (cryptogenic or of known cause) and in the general population (healthy population or patients with diseases other than cerebrovascular disease), using autopsy or a validated ultrasound diagnostic modality (TEE, TTE, TCD). Patent foramen ovale documentation per diagnostic modality was as follows: (1) autopsy studies were conducted in patients with a cause of death other than cerebrovascular disease, and foramen ovale patency was demonstrated via a probe or a pencil; (2) in most TEE and TTE studies, investigations were evaluated by two different cardiologists and considered positive if one to five microbubbles were detected after the use of gelatin or saline contrast within three to five heart cycles after opacification of the right atrium, at rest and during Valsalva maneuver; (3) TCD examinations were also evaluated by one or two neurologists and considered positive if one to three microembolic signals were detected within 15–40 s after the injection of gelatin or saline contrast, at rest and during Valsalva maneuver.

Studies were included if (1) they reported data from a general population or from subjects of all ages without known cerebrovascular disease, who were referred for PFO detection; (2) they specified the etiologic type of ischemic cerebrovascular event as cryptogenic (CE) vs. event of known cause (non-CE); (3) they reported PFO prevalence in patients with transient ischemic attacks (TIAs) and stroke as a single group. In studies that separately reported PFO prevalence in patients with TIAs and stroke, only data from the latter were included in the analysis. Furthermore, we included data from studies in migraineurs that reported PFO prevalence in a non-migraineur population arm. Studies were excluded if (1) they reported data from subjects with an underlying cerebrovascular disease, who were referred for PFO detection; (2) they did not specify the type of ischemic cerebrovascular event. For duplicate studies, we included only the updated article with the most informative data. We did not include review articles of previously included studies unless new data were reported. The extracted information was stratified and analyzed by diagnostic modality (autopsy, TEE, TTE, TCD), health status (healthy population/controls vs. stroke), CS status (yes vs. no), and age (young vs. old per authors' definition). Patent foramen ovale prevalence between different age and diagnostic modality subgroups was compared using the χ^2^ test. For the included studies, we calculated odds ratios (ORs) for PFO prevalence in CE compared with healthy/control population and also compared with non-CE, individually and cumulatively, stratified by diagnostic modality.

## Results

Our search resulted in 1,032 studies, which were individually assessed. We identified 66 relevant articles, of which 54 were finally included in our review (Table 1) (16–69). We found 10 autopsy studies with 7,747 subjects (16–25). Patent foramen ovale was documented in 1,872 of them [24.2%, 95% confidence interval (CI) = 23.2–25.1]. We included 26 TEE studies in total (26–51). One study (29) was exclusively conducted on a healthy population. One study was conducted on a healthy population compared with migraineurs with aura (35). Twenty-four studies reported data from patients with cerebrovascular ischemic events (CE or non-CE), of which four studies also included TIAs (10–20% of the total events) (2, 31, 48, 49). Three studies also investigated a healthy population (27, 28, 34), and five studies also investigated control patients who underwent TEE for reasons other than ischemic cerebrovascular events (26, 30–33). Cumulatively, PFO was documented in 325 of 1,369 (23.7, 95% CI = 21.6–26.1) healthy subjects/controls, in 1,630 of 4,097 (39.8, 95% CI = 38.3–41.3) patients with CE and in 281 of 1,329 (21.1, 95% CI = 19.0–23.4) patients with non-CE. We included six TTE studies (52–57). One study was exclusively conducted on a healthy population (53). One study was conducted on a healthy population compared with migraineurs (54). Four studies (52, 55–57) reported data from patients with cerebrovascular ischemic events, of which one study also included TIAs in unknown percentage (55). One study (55) also investigated a healthy population, and one study (52) also investigated patients without cerebrovascular events who underwent TTE as a preparation for posterior fossa surgery. Cumulatively, PFO was documented in 186 of 1,267 (14.7, 95% CI = 12.8–16.7) healthy subjects/controls, in 66 of 131 (50.4, 95% CI = 41.9–58.8) patients with CE, and in 11 of 125 (8.8, 95% CI = 4.8–15.2) patients with non-CE. In our review, we included 12 TCD studies (58–69). Three studies were conducted in migraineurs compared to a healthy population (59–61), and nine studies reported data from patients with cerebrovascular events, of which five studies (63–67) also included TIAs (20–75% of the total events). Two studies also investigated a healthy population (58, 62). Cumulatively, PFO was documented in 111 of 355 (31.3, 95% CI = 26.7–36.3) healthy subjects/controls, in 706 of 1,591 (44.4, 95% CI = 41.9–46.8) patients with CE, and in 323 of 1,516 (21.3, 95% CI = 19.3–23.4) patients with non-CE.

**Table 1:** List of included studies and PFO prevalence by diagnostic modality.

**Studies—all ages**	**Healthy/control population**	**Cryptogenic events**	**Non-cryptogenic events**	
	**PFO(+)/Total Prevalence (%)**	**PFO(+)/Total Prevalence (%)**	**PFO(+)/Total Prevalence (%)**	**Age (years)**
**Autopsy Studies**				
Hagen et al. (16)	263/965			>1
	27.2			
Thompson and Evans (17)	386/1,100			All
	35.1			
Patten (18)	683/3,277			Mostly adults
	20.8			
Parsons and Keith (19)	103/399			All
	25.8			
Fawcett and Blachford (20)	96/306			>10
	31.4			
Seib (21)	85/500			>20
	17			
Wright et al. (22)	113/492			Mostly adults
	23			
Schroeckenstein et al. (23)	50/144			>20
	34.7			
Sweeney and Rosenquist (24)	20/64			>10
	31.2			
Penther (25)	73/500			Adults
	14.6			
**Total**	**1,872/7,747**			
	**24.2**			
**Transesophageal Echocardiography**				
Cabanes et al. (26)	9/50	36/64	7/36	<55
	18	56.3	19.5	
Jones et al. (27)	2/19	4/14	3/12	<50
	11	28.6	25	
	18/117	5/30	10/59	50–69
	15	16.7	17	
	11/66	5/27	8/78	>70
	17	18.5	10.2	
Job et al. (28)	27/63	27/41	11/33	<55
	43	65.8	33.3	
Meissner et al. (29)	148/581			>45
	25.5			
Mesa et al. (30)	7/35	23/55		<50
	20	42		
Cerrato et al. (31)	6/27	27/53		<50
	22.2	51		
	7/51	16/53		>50
	13.7	30.1		
Hausmann et al. (32)	2/18	9/18	0/1	<40
	11.1	50	0	
	23/98	3/20	6/22	>40
	23.5	15	27.3	
van Camp et al. (33)	11/28	19/24		All
	39.3	79.1		
Schuchlenz et al. (34)	38/123	54/66		<60
	30.9	81.8		
Schwerzmann et al. (35)	16/93			Young
	17.2			
Ranoux et al. (36)		31/54	1/14	<55
		57.4	7.1	
Homma et al. (37)		16/36	7/38	All
		44.4	18.4	
Petty et al. (38)		22/55	15/61	All
		40	25	
Mas et al. (39)		267/581		<55
		46		
Homma et al. (40)		98/250	105/351	All
		39.2	29.9	
Petty et al. (41)		33/133	27/158	All
		24.8	17.1	
Handke et al. (42)		36/82	7/49	<55
		43.9	14.3	
		41/145	27/227	>55
		28.3	11.9	
Zahn et al. (43)		50/118	18/70	All
		42.4	25.7	
Di Tullio et al. (44)		9/19	8/25	All
		47.3	32	
Kim et al. (45)		76/245		All
		31		
Komar et al. (46)		69/88		<55
		78.4		
De Castro et al. (47)		133/343		All
		38.8		
Weimar et al. (48)		376/1,126		All
		33.4		
Nighoghossian et al. (49)		27/79		<60
		34		
Klötzsch et al. (50)		31/40	19/71	All
		77.5	26.7	
Mesa et al. (51)		70/194	2/24	<55
		36	8.3	
		17/44		>55
		38.6		
**Total**	**325/1369**	**1,630/4,097**	**281/1,329**	
	**23.7**	**39.8**	**21.1**	
**Transthoracic Echocardiography**				
Lechat et al. (52)	10/100	20/41	4/19	<55
	10	48.8	21	
Di Tullio et al. (53)	164/1,100			>39
	14.9			
Tatlidere et al. (54)	6/27			All
	22.2			
Webster et al. (55)	6/40	19/34		<40
	15	55.9		
Di Tullio et al. (56)		10/21	1/24	<55
		47.6	4.2	
		9/24	6/77	>55
		37.5	7.8	
Jeanrenaud et al. (57)		8/11	0/5	<50
		72.7	0	
**Total**	**186/1,267**	**66/131**	**11/125**	
	**14.7**	**50.4**	**8.8**	
**Transcranial Doppler**				
Serena et al. (58)	32/100	30/53	38/150	All
	32	56.6	25.3	
Del Sette et al. (59)	8/50			<50
	16			
Anzola et al. (60)	5/25			<55
	20			
Domitrz et al. (61)	16/65			<55
	24.6			
Koutroulou et al. (62)	50/115	42/84		<55
	43.5	50		
Serena et al. (63)		162/229		<55
		70.7		
		135/257		>55
		52.5		
Mazzuco et al. (64)		29/74	16/52	<60
		39.2	30.8	
		68/190	44/207	>60
		35.8	21.2	
Palazzo et al. (65)		34/47		<55
		72.3		
Yeung et al. (66)		16/27		<50
		59.3		
		27/89		>50
		30.3		
			17/94	All
			18	
Schminke et al. (67)		33/60	8/40	All
		55	20	
Consoli et al. (68)		77/327	170/797	All
		23.5	21.3	
Carod-Artal et al. (69)		37/90	5/40	<45
		41.1	11.1	
		16/64	25/136	>45
		25	18.4	
**Total**	**111/355**	**706/1,591**	**323/1,516**	
	**31.3**	**44.4**	**21.3**	

*All ages are included*.

Tables 2, 3 present the results of our review in young and old subjects, respectively. The age cutoff per individual study ranged between 40 and 60 years. In healthy/control population, there was no difference of PFO prevalence between the young and the old age groups, when the diagnostic modality was TEE (25 vs. 22.7%, *p* = 0.35) or TTE (11.4 vs. 14.9%, *p* = 0.07). Concerning TCD, a comparison was not possible because data were not available for the old age group. In patients with CE, PFO prevalence in the young compared to the old age group was higher when the diagnostic modality was TEE (48.9 vs. 27.3%, *p* < 0.0001, OR = 2.6 with 95% CI = 2.0–3.3) or TCD (58.1 vs. 41%, *p* < 0.0001, OR = 1.9 with 95% CI = 1.6–2.5), but not TTE (53.3 vs. 37.5%, *p* = 0.16). Finally, in patients with non-CE, PFO prevalence in the young compared to the old age group was higher when the diagnostic modality was TEE (20.0 vs. 12.9%, *p* = 0.04, OR = 1.7 with 95% CI = 1.0–2.8) but not TTE (10.4 vs. 7.8%, *p* = 0.75) or TCD (22.8 vs. 20.1%, *p* = 0.56).

**Table 2:** List of included studies and PFO prevalence by diagnostic modality in the young.

**Studies—young**	**Healthy/control population**	**Cryptogenic events**	**Non-cryptogenic events**	**Age (years)**
	**PFO(+)/Total Prevalence (%)**	**PFO(+)/Total Prevalence (%)**	**PFO(+)/Total Prevalence (%)**	
**Transesophageal Echocardiography**				
Cabanes et al. (26)	9/50	36/64	7/36	<55
	18	56.3	19.5	
Jones et al. (27)	2/19	4/14	3/12	<50
	11	28.6	25	
Job et al. (28)	27/63	27/41	11/33	<55
	43	65.8	33.3	
Mesa et al. (30)	7/35	23/55		<50
	20	42		
Cerrato et al. (31)	6/27	27/53		<50
	22.2	51		
Hausmann et al. (32)	2/18	9/18	0/1	<40
	11.1	50	0	
Schuchlenz et al. (34)	38/123	54/66		<60
	30.9	81.8		
Schwerzmann et al. (35)	16/93			Young
	17.2			
Ranoux et al. (36)		31/54	1/14	<55
		57.4	7.1	
Mas et al. (39)		267/581		<55
		46		
Handke et al. (42)		36/82	7/49	<55
		43.9	14.3	
Komar et al. (46)		69/88		<55
		78.4		
Nighoghossian et al. (49)		27/79		<60
		34		
Mesa et al. (51)		70/194		<55
		36		
**Total**	**107/428**	**680/1,389**	**29/145**	
	**25**	**48.9**	**20**	
**Transthoracic Echocardiography**				
Lechat et al. (52)	10/100	20/41	4/19	<55
	10	48.8	21	
Webster et al. (55)	6/40	19/34		<40
	15	55.9		
Di Tullio et al. (56)		10/21	1/24	<55
		47.6	4.2	
Jeanrenaud et al. (57)		8/11	0/5	<50
		72.7	0	
**Total**	**29/140**	**57/107**	**5/48**	
	**11.4**	**53.3**	**10.4**	
**Transcranial Doppler**				
Del Sette et al. (59)	8/50			<50
	16			
Anzola et al. (60)	5/25			<55
	20			
Domitrz et al. (61)	16/65			<55
	24.6			
Koutroulou et al. (62)	50/115	42/84		<55
	43.5	50		
Serena et al. (63)		162/229		<55
		70.7		
Mazzuco et al. (64)		29/74	16/52	<60
		39.2	30.8	
Palazzo et al. (65)		34/47		<55
		72.3		
Yeung et al. (66)		16/27		<50
		59.3		
Carod-Artal et al. (69)		37/90	5/40	<45
		41.1	11.1	
**Total**	**79/255**	**320/551**	**21/92**	
	**31**	**58.1**	**22.8**	

**Table 3:** List of included studies and PFO prevalence by diagnostic modality in the old.

**Studies—old**	**Healthy/control population**	**Cryptogenic events**	**Non-cryptogenic events**	**Age (years)**
	**PFO(+)/Total Prevalence (%)**	**PFO(+)/Total Prevalence (%)**	**PFO(+)/Total Prevalence (%)**	
**Transesophageal Echocardiography**				
Jones et al. (27)	29/183	10/57	18/137	>50
	15.8	17.5	13.1	
Meissner et al. (29)	148/581			>45
	25.5			
Cerrato et al. (31)	7/51	16/53		>50
	13.7	30.1		
Hausmann et al. (32)	23/98	3/20	6/22	>40
	23.5	15	27.3	
Handke et al. (42)		41/145	27/227	>55
		28.3	11.9	
Mesa et al. (51)		17/44	2/24	>55
		38.6	8	
**Total**	**207/913**	**87/319**	**53/410**	
	**22.7**	**27.3**	**12.9**	
**Transthoracic Echocardiography**				
Di Tullio et al. (53)	164/1,100			>39
	14.9			
Di Tullio et al. (56)		9/24	6/77	>55
		37.5	7.8	
**Total**	**164/1,100**	**9/24**	**6/77**	
	**14.9**	**37.5**	**7.8**	
**Transcranial Doppler**				
Serena et al. (63)		135/257		>55
		52.5		
Mazzuco et al. (64)		68/190	44/207	>60
		35.8	21.2	
Yeung et al. (66)		27/89		>50
		30.3		
Carod- Artal et al. (69)		16/64	25/136	>45
		25	18.4	
**Total**		**246/600**	**69/343**	
		**41**	**20.1**	

**Figure 1:**
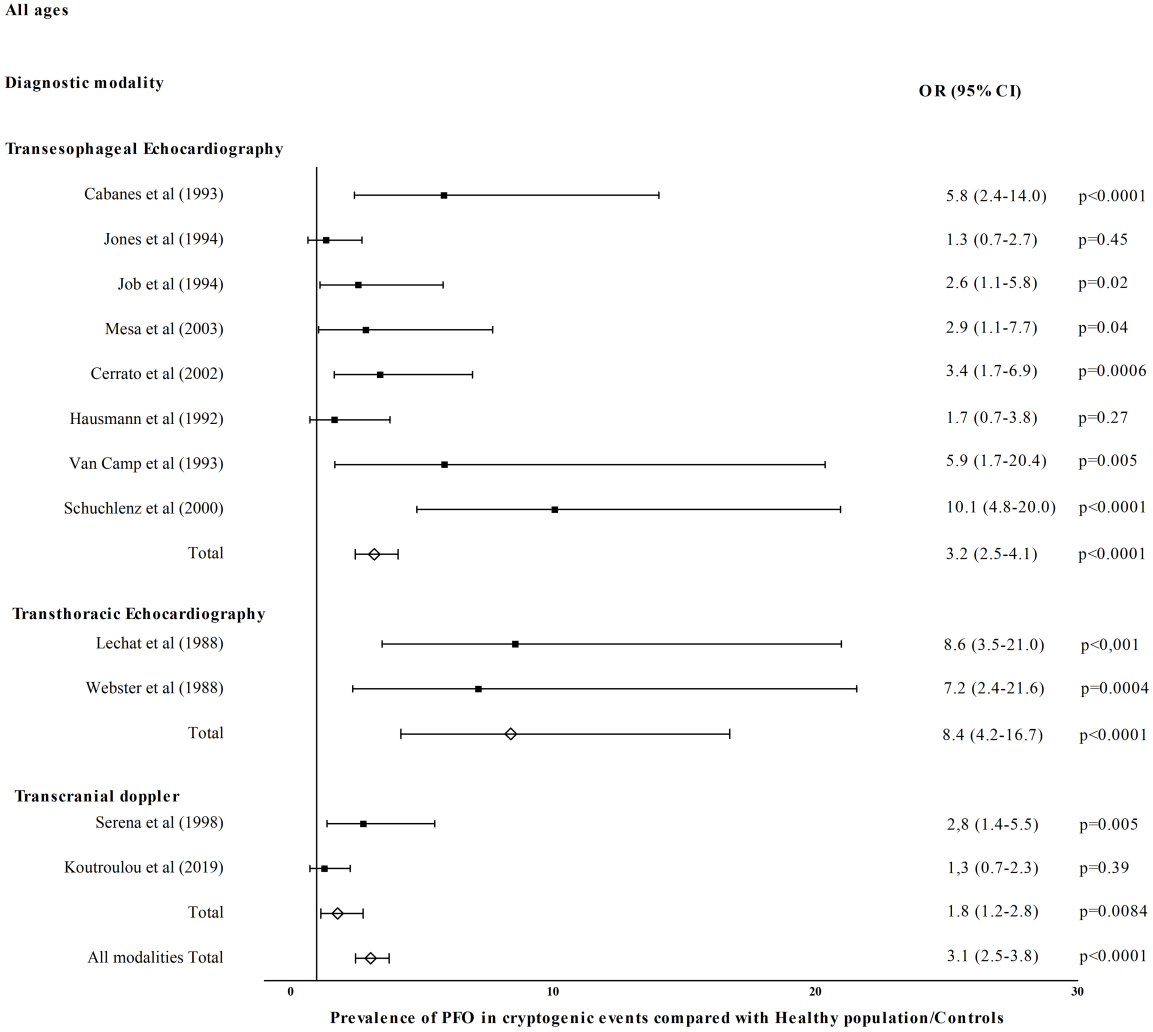
Prevalence of PFO in cryptogenic events compared with healthy population/controls.

**Figure 2:**
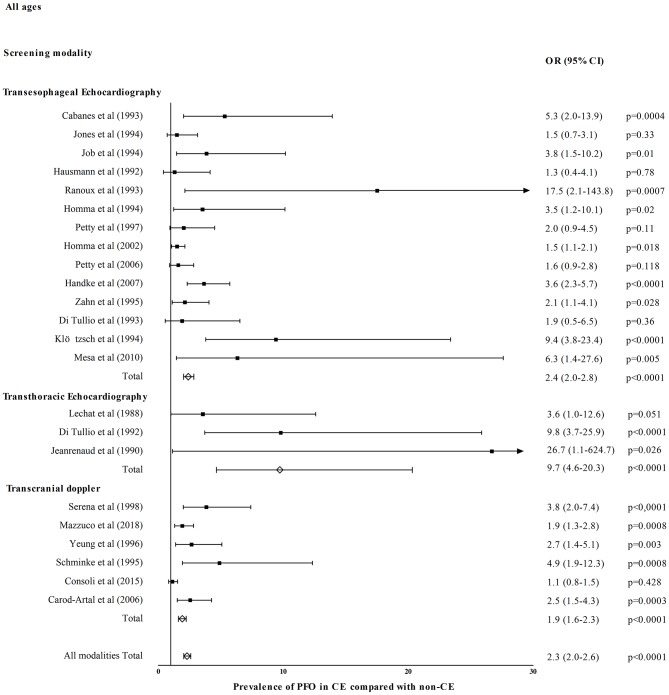
Prevalence of PFO in cryptogenic events (CE) compared with events of known cause (non-CE).

Figure 1 shows OR for PFO prevalence in CE compared with healthy/control population for eight TEE studies (26–28, 30–34), two TTE studies (52, 55), and two TCD studies (58, 62). Patent foramen ovale prevalence was higher in CE in TEE (OR = 3.2, 95% CI = 2.5–4.1, *p* < 0.0001), TTE (OR = 8.4, 95% CI = 4.2–16.7, *p* < 0.0001), and TCD studies (OR = 1.8, 95% CI = 1.2–2.8, *p* = 0.008). All diagnostic modalities included PFO prevalence was higher in CE compared with healthy/control population (OR = 3.1, 95% CI = 2.5–3.8, *p* < 0.0001). Figure 2 shows OR for PFO prevalence in CE compared with non-CE for 14 TEE studies (26–28, 32, 36–38, 40–44, 50, 51), three TTE studies (52, 56, 57), and six TCD studies (58, 64, 66–69). Patent foramen ovale prevalence was higher in patients with CE in TEE (OR = 2.4, 95% CI = 2.0–2.8, *p* < 0.0001), TTE (OR = 9.7, 95% CI = 4.7–20.3, *p* < 0.0001), and TCD studies (OR = 1.9, 95% CI = 1.6–2.3, *p* < 0.0001). All diagnostic modalities included, PFO prevalence was higher in CE compared with non-CE (OR = 2.3, 95% CI = 2.0–2.6, *p* < 0.0001).

## Discussion

Patent foramen ovale is not rare in the general population, but its detection has increasingly gained interest during the last two centuries, especially after its association with paradoxical embolism. Until late twentieth century, PFO detection relied exclusively on autopsy studies owing to lack of accurate *in vivo* diagnostic methods. However, even the more recent and better conducted studies admitted inherent limitations such as the use of formalin-fixed and not fresh specimens (16). The latter could have limited the detection of small-to-medium interatrial patency due to shrinkage of the fixed fibroelastic elements of the foramen ovale. Further possible disadvantages included the use of probes that could identify PFOs only larger than 1 mm and the inclusion of children. Interestingly, Hagen et al. (16) observed that PFO incidence was higher in younger subjects; conversely, PFO size was bigger in older subjects. They hypothesized that the former may be attributed to the increasing incidence of spontaneous anatomic closure of relatively small PFOs with advancing age, caused by age-related fibroelastic thickening of the valve of fossa ovalis. Consequently, relatively larger PFOs remain in late adult life, and their size may undergo further modification by stretching (16).

The development of echocardiography (initially TTE and later TEE) during the second half of the twentieth century provided the first *in vivo* diagnostic tools for PFO. A second breakthrough in PFO detection happened after the development of TCD by Aaslid et al. (70) in 1982. Etiologic classification systems of ischemic stroke consider PFO as a medium-to-low or uncertain-risk emboligenic cardiac source (71, 72). Accordingly, the latest RCTs (10–12) documented spectacular superiority of percutaneous PFO closure only in carefully selected patients with CSs over best medical treatment, hence the need to detect reliably PFO in CS sufferers with the three available ultrasound modalities. Hitherto, TEE is considered the “gold standard” for the documentation of PFO (73, 74). A meta-analysis comparing TTE with TEE as a reference in 3,067 patients (75) evidenced the low sensitivity (45.1%) but very high specificity (99.6%) of TTE for PFO detection. The former can be attributed to several technical limitations: (1) atrial structures are located in the far ultrasound beam field and are subjected to acoustic interference by the chest wall; (2) during right-to-left shunt (RLS) provoking maneuvers, there is considerable lung interference, interrupting continuous imaging of the atria; (3) there is limited ability to document increased right-to-left atrial pressure gradient by visualizing movement of the septum toward the left atrium (73). Consequently, TTE even when performed with contrast agent and RLS provoking maneuvers is a poor screening tool for PFO: a negative examination should not rule out PFO presence, particularly if clinical suspicion is high.

The potentially causal relationship of PFO with some of cryptogenic ischemic events of the brain led vascular neurologists to incorporate contrast TCD in their routine workup for CS for more than 20 years, especially after the standardization of the technical protocol for the detection and quantification of RLS (76). Transcranial Doppler lacks direct visualization of atrial structures and documents RLS regardless of the subjacent pathology: PFO or (rarely) pulmonary arteriovenous malformations (PAVMs). However, it is the only diagnostic modality that (1) proves the emboligenic potential of RLS to the target organ (brain) and (2) quantifies the burden of embolism (number of microembolic signals corresponding to microbubbles) to the recipient (brain) and not to the source (left atrium). Furthermore, TCD is non-invasive, safe, and easily repeatable with low cost, and patients are alert and able to perform effective and calibrated Valsalva maneuvers. The latter may have significant impact on shunt quantification (77) and represents a major limitation of TEE because patients tend to perform ineffective Valsalva maneuvers owing to poor cooperation under sedation, dysphagia, or to the presence of the TEE probe in their esophagus. Additionally, TEE has certain esophagus-related contraindications (varices, diverticula, strictures, Barrett esophagus, Mallory-Weiss tear, important hemorrhagic risk) and may have rare but severe complications (aspiration, esophageal bleeding, or perforation).

Meta-analyses comparing TCD with TEE (75, 78) concluded that TCD has excellent diagnostic accuracy and should be used as a first-choice screening tool for PFO in patients with CS, reserving TEE to provide complementary anatomic details that may influence treatment decisions (PFO morphology, presence of atrial septum aneurysm). An updated meta-analysis of 2,751 patients by the authors of the European position paper on the management of patients with PFO (79) reconfirmed the excellent accuracy of TCD compared with TEE (sensitivity of 94%, specificity of 92%, area under the receiver operating characteristic curve of 0.97). Although TEE has been considered as the “gold standard” for PFO detection, there is good evidence to think that TEE is a standard of uncertain validity. Most of the studies that compared the two modalities did not verify the origin of presumed false-positive TCD results. Frequently, the latter were arbitrarily attributed to possible PAVMs, an entity considered particularly rare with a prevalence of 1 in 2,600 (80). Furthermore, PAVMs may sometimes be misinterpreted by TEE as well (78). A meta-analysis of 164 patients comparing TEE with autopsy, cardiac surgery, and/or catheterization as the gold standard showed a sensitivity of 89.2% and specificity of 91.4% to detect PFO and concluded that TEE should be complemented by highly sensitive screening tests, namely, TCD (81). Estimation of the degree of RLS in all patients undergoing cardiac catheterization for PFO closure could be used as an alternative gold standard and could be compared with preprocedural TEE and TCD data. The superior sensitivity of TCD has also been demonstrated in a study (82) where TEE failed to document RLS in 15% of patients with CS, and of those, 40% had large RLSs. Therefore, “false-positive” TCD results may, in fact, represent true PFOs that are missed because of TEE limitations, and a negative TEE should not negate the need for a complementary TCD investigation.

According to our review, PFO prevalence in the general population across all ages was roughly 24% in autopsy and TEE studies. As expected, this percentage was much smaller in TTE studies (15%), whereas in the highly sensitive TCD studies, PFO prevalence was higher (~31%). The results were similar with small differences when subjects were stratified into young and old age groups. The results should be viewed under the limitations of the relatively small size (355 subjects) of healthy population in TCD studies and of the absence of TCD data in the old age group. Future TCD studies should focus on elderly general population and provide evidence regarding the differential PFO prevalence and magnitude of RLS with increasing age, as suggested by autopsy studies. Furthermore, in three of five TCD studies that estimated PFO prevalence (59–61), the healthy population comprised non-migraineurs, resulting in prevalence as low as 16% (59). Because migraineurs constitute 10–15% (83) of the general population and migraineurs are more likely to have a PFO (84), future studies on PFO prevalence in the general population should not exclude migraineurs.

Of note is the considerable variability in PFO prevalence among studies that used the same diagnostic modality. In autopsy studies, PFO prevalence ranged from 14.6 to 35.1%, in TEE studies from 11 to 43%, in TTE from 10 to 22.2%, and in TCD studies from 16 to 43.5%. The heterogeneous results could be attributed to (1) selection bias because in most ultrasound-based studies the reported “healthy population” consisted of patients who underwent an examination for a reason other than cerebrovascular event, and PFO detection was not the primary endpoint; (2) technical differences in PFO detection and RLS quantification; (3) different PFO prevalence in discrete ethnic/racial populations. Hitherto, the latter issue has not been addressed, and a “fixed” 25% (mainly based on autopsy and TEE studies) has been established as PFO prevalence across the general population and has been used for the calculation of PFOAF (15). However, given the limitations of autopsy and TEE studies, there is good reason not to take this percentage for granted. Interestingly, a recent TCD study conducted in a national population that comprised healthy Greek adults younger than 55 years and included subjects with migraine without aura (~10% of the total population) found much higher PFO prevalence (43.5%) compared to previous TCD studies in other populations (62). Interest in optimal patient selection for PFO closure or possibly for long-term anticoagulation with direct oral anticoagulants (85) remains keen and the RoPE score may be useful in guiding patient management; albeit it lacks large external validation studies, and it is heavily age weighted. Therefore, the estimation of degree of causality (PFOAF) may be underestimated or overestimated in ethnic/racial populations with PFO prevalence significantly lower or higher than the established 25%.

Although this review is not systematic and does not include meta-analytic methodology, it has the advantage of including only studies with a clear etiologic classification of stroke (cryptogenic vs. non-cryptogenic). We excluded studies with a vague definition of CS or studies that included “pseudo” CSs, and we excluded data from patients with TIAs. Transient ischemic attacks are a “soft” and overused diagnosis, and TIA definition has evolved over the years from time-specific to tissue-specific (86). Reversible deficits, particularly in the elderly, may be caused by amyloid angiopathy, an easily missed diagnosis unless blood-sensitive magnetic resonance imaging sequences are performed (87). Accordingly, all recent successful PFO closure trials did not include patients with TIAs (10–12).

In our review, PFO prevalence was nearly 2-fold in CE compared with non-CE (OR ranging widely from 1.1 to 17.5 in individual studies) in accordance with previous random-effects meta-analyses that established the strong association between CS and PFO with OR in the order of 2.9 (88, 89). This marked difference persisted regardless of age confirming a meta-analysis in older patients with OR in the order of 2.5 (64). However, young patients with CE had higher PFO prevalence compared to older patients reflecting the stronger association of CE with PFO in younger ages (88, 89). Concerning non-CE, PFO prevalence across the board and particularly in older patients was numerically lower than in the general population possibly owing to the decreasing frequency and less implication of PFO in stroke mechanisms with increasing age (16). We showed that PFO prevalence across all ages was ~3-fold in CE compared with healthy population/controls with OR ranging from 1.3 to 10.1. This is in accordance with random-effects OR from previous meta-analyses ranging from 2.1 to 2.9 (88, 89). The above association is mainly driven by TEE and TTE studies, whereas only two TCD studies compared PFO prevalence in CE with a relatively small non-selected general population of 215 subjects in total (58, 62). Given the high sensitivity, non-invasive nature, low cost, and repeatability of TCD, future large-scale TCD-based studies should investigate potential heterogeneity in PFO prevalence in different healthy racial/ethnic populations. The latter may have important implications in individualizing PFO-associated stroke risk assessment and management in the forthcoming era of precision medicine.

## Author Contributions

IK and TK data acquisition, data analysis and interpretation, drafting of the manuscript. GT data interpretation, critical revision for important intellectual content. DT data acquisition, data analysis, and interpretation. DK and NG critical revision for important intellectual content.

## Conflict of Interest

The authors declare that the research was conducted in the absence of any commercial or financial relationships that could be construed as a potential conflict of interest.
